# Palmitoleic acid sensitizes vancomycin-resistant *Staphylococcus aureus* to vancomycin by outpacing the expression of resistance genes

**DOI:** 10.1128/spectrum.01996-24

**Published:** 2024-12-10

**Authors:** Zajeba Tabashsum, Michelle Angeles-Solano, Ashelyn E. Sidders, Joshua B. Parsons, Sarah E. Rowe

**Affiliations:** 1 Department of Microbiology and Immunology, University of North Carolina-Chapel Hill, Chapel Hill, North Carolina, USA; 2 Division of Infectious Diseases, Duke University School of Medicine, Durham, North Carolina, USA; University of Saskatchewan, Saskatoon, Saskatchewan, Canada

**Keywords:** antibiotic resistance, *Staphylococcus aureus*, antibiotic adjuvants

## Abstract

The rise in antibiotic resistance limits the availability of antibiotics to treat bacterial infections. Despite this, antibiotic development pipelines remain sparse which makes using adjuvants to reverse antibiotic resistance a promising therapeutic strategy. The use of vancomycin, a frontline antibiotic used to treat hospitalized patients with methicillin-resistant *Staphylococcus aureus* (MRSA) infections, is complicated by high rates of treatment failure. Vancomycin binds to the D-ala-D-ala terminus of the nascent peptidoglycan precursor lipid II, preventing cell wall biosynthesis. Vancomycin-resistant strains of *S. aureus* and *Enterococci* typically express a *van* gene cluster that is induced in response to vancomycin and results in the synthesis of an alternative lipid precursor with a peptide chain ending in D-ala-D-lac. Vancomycin has low affinity for the D-ala-D-lac terminus, and the bacteria can resume growth even in the presence of an otherwise lethal dose of vancomycin. We previously showed that palmitoleic acid, a host-produced monounsaturated fatty acid, combined with vancomycin led to an accumulation of large fluid patches in the bacterial membrane, resulting in membrane destabilization and cell death. In this study, we observed that palmitoleic acid increases the rate of vancomycin killing by more than 50-fold, compared to vancomycin alone. This rapid bactericidal activity by the combined treatment sensitized vancomycin-resistant *S. aureus* (VRSA) and vancomycin-resistant *Enterococcus* (VRE) to vancomycin, likely by outpacing the expression of vancomycin resistance genes. This study represents an important step in the ongoing effort to mitigate antibiotic resistance.

**IMPORTANCE:**

The development of antibiotics has transformed medicine, reducing the incidence and severity of bacterial infections and allowing for advancements in healthcare, including invasive surgeries and organ transplants. However, the rise of antibiotic resistance poses a significant threat to these medical advancements, leading to treatment failures that result in increased patient morbidity and mortality, as well as substantial healthcare costs. Vancomycin-resistant *Enterococcus* (VRE) species are prevalent in hospital settings and chronic infections. Although high-level vancomycin resistance in *S. aureus* is rare, *S. aureus* can acquire plasmids expressing vancomycin resistance genes from resistant Enterococcal species during infection, further complicating treatment. In this study, we find that palmitoleic acid increases the rate of vancomycin killing and restores sensitivity to vancomycin-resistant *S. aureus* (VRSA) and VRE isolates.

## OBSERVATION

Vancomycin is a glycopeptide antibiotic with activity against Gram positive bacteria including *Staphylococcus aureus*, *Clostridiodes difficile,* and *Enterococcus* species (1). Vancomycin binds to the membrane-bound D-ala-D-ala terminus of the cell wall precursor lipid II which prevents its incorporation into the expanding peptidoglycan layer (2). This steric hindrance causes an accumulation of lipid II on the cell surface and halts cell wall biosynthesis resulting in cell death (1, 2). The bactericidal effect of vancomycin is primarily against cells that are actively dividing, but its activity is limited against non-growing cells and persister cells (3).

High-level resistance to vancomycin occurs due to the acquisition of a *van* gene cluster that typically encodes seven proteins. These proteins participate in one of three functions: (1) the VanRS two-component regulatory system that senses vancomycin and regulates the expression of resistance (VanS is the histidine kinase and VanR the response regulator of the system), (2) enzymes (VanH, a dehydrogenase and VanA, an amino acid ligase) which alter the terminal peptide of lipid II from D-ala-D-ala to D-ala-D-lac, which has ~1,000 fold reduced affinity for vancomycin (4, 5), or (3) VanX and VanY enzymes that remove the native D-ala-D-ala-ending precursors.

Vancomycin-resistant *Enterococcus* (VRE) is a significant concern in hospital environments. In contrast, vancomycin-resistant *S. aureus* (VRSA) strains are considered rare with only 14 cases identified in the USA to date (1, 5). The majority of these strains have been isolated from diabetic patients suffering from chronic wounds that are co-infected with VRE and *S. aureus* (5). Importantly, these VRSA isolates have arisen in clonal complexes typically associated with hospital acquisition and have not demonstrated the ability to spread from person to person (6, 7). However, a report from Brazil described a patient with bacteremia caused by a community-acquired methicillin-resistant *S. aureus* (CA-MRSA) isolate that acquired a vancomycin resistance plasmid during vancomycin therapy (6). CA-MRSA strains are known to have increased capacity for dissemination and virulence compared to hospital-acquired isolates (8). The convergence of CA-MRSA to VRSA underscores the importance of developing novel therapeutic strategies to overcome vancomycin resistance.

We have previously shown that palmitoleic acid improves vancomycin efficacy, and this novel drug combination has potent activity against persister cells and non-growing cells (7). Palmitoleic acid is a 16-carbon monounsaturated fatty acid which permeabilizes the membrane of Gram-positive bacteria (9). Palmitoleic acid in combination with vancomycin led to the accumulation of membrane-bound cell wall intermediates which generated large regions of increased fluidity (RIFs), septal disorganization, and subsequent cell death (7). We also showed that palmitoleic acid re-sensitizes VRSA strains to vancomycin through an unknown mechanism (7). In this study, we examine the mechanism by which palmitoleic acid overcomes vancomycin resistance in VRSA and VRE isolates.

### Palmitoleic acid potentiates vancomycin efficacy against VRSA and VRE strains

**Fig 1:**
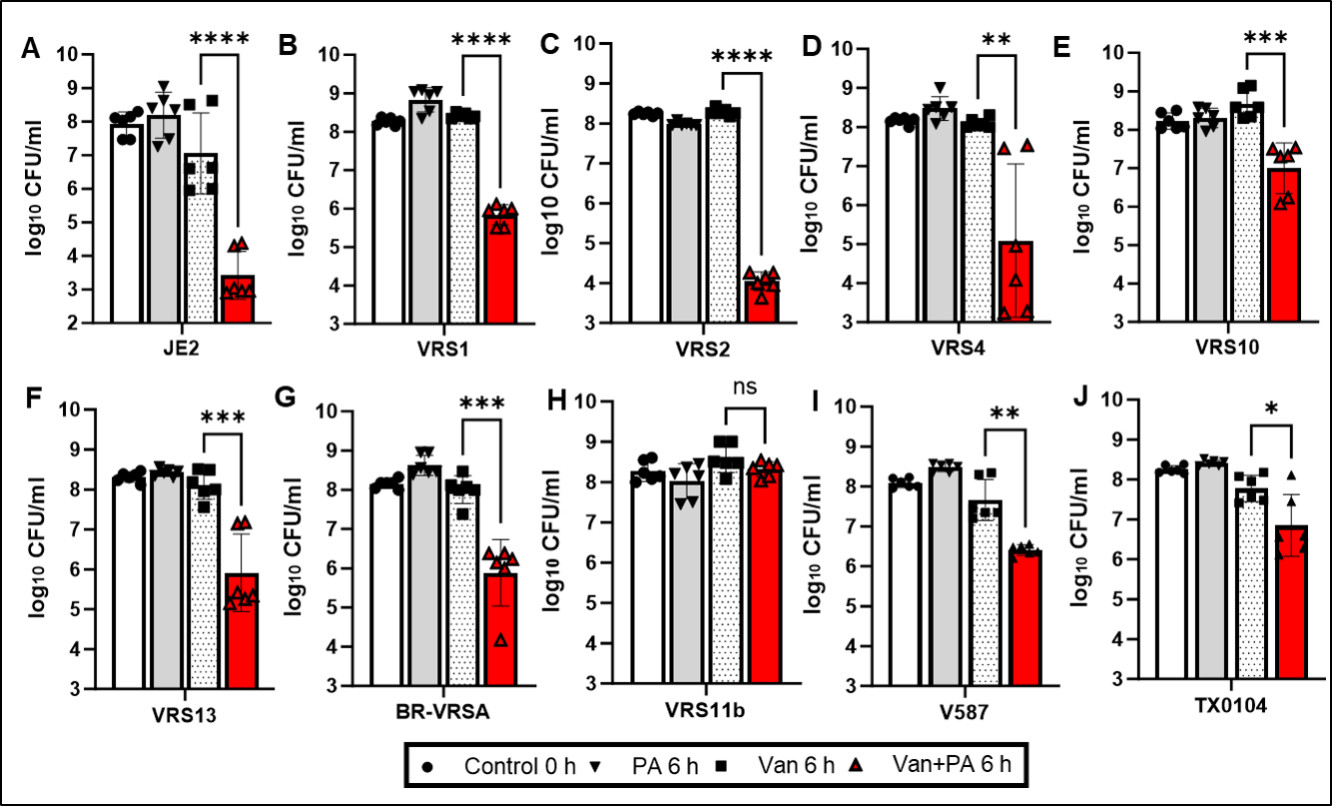
Palmitoleic acid enhances vancomycin killing of VRSA and VRE. (**A**) Vancomycin sensitive strain *S. aureus* (VSSA) JE2 and (**B–H**) VRSA and (**I and J**) VRE were cultured to mid-exponential phase in tryptic soy broth (TSB) and treated with 30 µg/mL palmitoleic acid and/or 50 µg/mL vancomycin (*C*_max_) for 6 h. Bacterial survivors were enumerated by plating. Data represent the mean value of *n = 6* biological replicates ± standard deviation. Statistical significance was determined by unpaired student *t*-test and denoted as ns, **, ***, or **** which represent not significant *P* > 0.05, *P* < 0.01, *P* < 0.001, and *P* < 0.0001, respectively.

We acquired seven VRSA and two VRE clinical isolates from the Biodefense and Emerging Infections Research Resources Repository (BEI) (10). These strains were grown to mid-exponential phase and challenged with the maximal achievable concentration of vancomycin in human serum (*C*_max_) (11) and/or a sub-MIC concentration of palmitoleic acid (Fig. 1; Table 1). Palmitoleic acid potentiated vancomycin efficacy against the vancomycin-sensitive *S. aureus* (VSSA) strain JE2 by 99.96% (Fig. 1A), which is consistent with our previous study (7). Next, we examined the efficacy of this combination treatment against the panel of VRSA and VRE isolates. Palmitoleic acid sensitized six out of the seven VRSA isolates and both VRE isolates to vancomycin (Fig. 1B through H). Palmitoleic acid increased vancomycin killing by 75%–99.99% (Table 1). To examine if the level of potentiation observed in each strain correlated to vancomycin susceptibility, we performed minimum inhibitory concentration (MIC) testing for each strain. The MIC of VRSA strains varied from 16 to 1,024 μg/mL (Table 1), but all strains were challenged with the same clinically achievable concentration of vancomycin (*C*_max_ 50 µg/mL), indicating that there was no association between the vancomycin MIC and the level of vancomycin potentiation.

**TABLE 1:** Minimum inhibitory concentrations of vancomycin and palmitoleic acid

Strain	Vancomycin MIC (µg/mL)	Palmitoleic acid MIC (µg/mL)	% increase in vancomycin killing in the presence of palmitoleic acid
JE2	1.0	16.0	99.96
VRS1	512.0	16.0	99.70
VRS2	16.0	16.0	99.99
VRS4	256.0	16.0	92.50
VRS10	256.0	32.0	95.76
VRS13	256.0	32.0	90.94
BR-VRSA	512.0	32.0	97.47
VRS11b	1,024.0	16.0	35.93
V587	32.0	32.0	90.46
TX0104	256.0	32.0	75.07

In the majority of VRSA isolates, the expression of the *van* resistance genes is induced by sensing vancomycin through the two-component system VanRS (12). However, in rare cases, the *vanHA* resistance genes are constitutively expressed even in the absence of vancomycin. Interestingly, the VRS11b isolate which constitutively expresses the *vanHA* resistant genes (13) was not re-sensitized to vancomycin by palmitoleic acid. In the inducible strains, the expression of the alternative cell wall precursors that are resistant to vancomycin likely takes some time after vancomycin sensing; therefore, we hypothesized that palmitoleic acid potentiates vancomycin killing by outpacing the expression of resistance genes.

**Fig 2:**
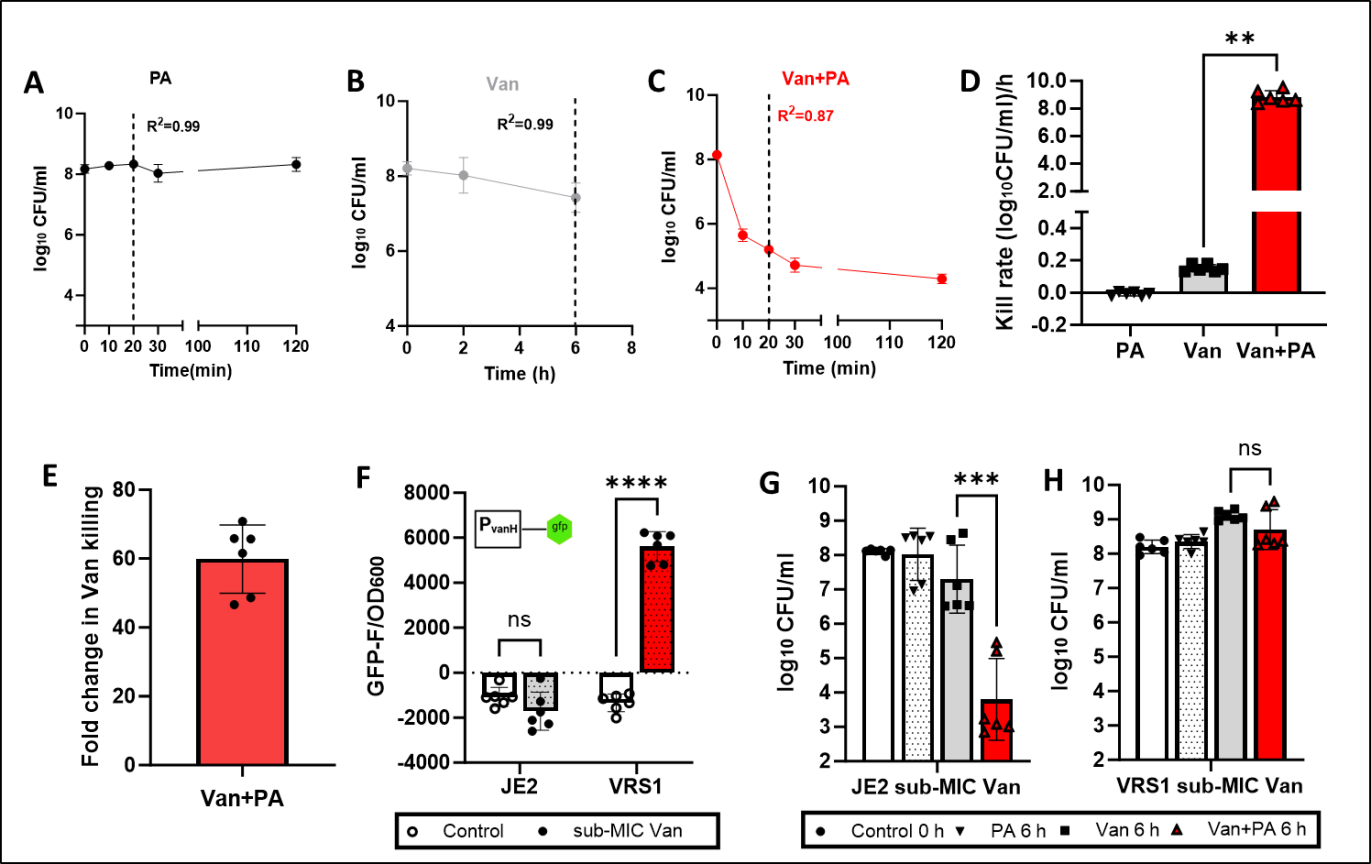
Palmitoleic acid increases the rate of vancomycin killing and outpaces expression of vancomycin resistance genes. *S. aureus* strain JE2 was cultured to mid-exponential phase in TSB and treated with (**A**) 30 µg/mL palmitoleic acid, (**B**) or 50 µg/mL vancomycin (*C*_max_), and (**C**) or the combination. At indicated times, survivors were enumerated by plating. (**D**) The rate of killing (log_10_ CFU_tN_ − log_10_ CFU_t0_)/Time was determined using the linear range of the assay (indicated by a vertical line in A–C). (**E**) Fold increase in the rate of vancomycin killing in the presence of palmitoleic acid. (**F**) Transcriptional activity of the *van* resistance genes was measured using a P*_vanH-gfp_* reporter plasmid in VRS1 and JE2 (negative control) following 3 h of exposure to sub-MIC vancomycin. (**G, H**) JE2 and VRS1 were grown to ~1 × 10^8^ CFU/mL in the presence of sub-MIC vancomycin before challenge with 30 µg/mL palmitoleic acid and/or 50 µg/mL vancomycin. After 6 h, bacterial survivors were enumerated by plating. Data represent the mean value of *n = 6* biological replicates ± standard deviation. Statistical significance was determined by unpaired *t*-test and denoted as ns, *, **, ***, and **** when *P* > 0.05, *P* < 0.05, *P* < 0.01, *P* < 0.001, and *P* < 0.0001, respectively.

To explore this, we assessed the rate of vancomycin killing with and without palmitoleic acid (Fig. 2A through E). Strikingly, palmitoleic acid increased the rate of vancomycin killing by ~58-fold (Fig. 2E). Next, we examined the ability of palmitoleic acid to potentiate vancomycin killing of a VRSA isolate after the conversion of D-ala-D-ala to D-ala-D-lac. Using a transcriptional reporter P*_vanH-gfp_*, we confirmed that sub-MIC vancomycin induced the expression of the *van* resistance genes in the VRSA strain (Fig. 2F). Additionally, pre-induction of the *van* resistance genes with sub-MIC vancomycin abrogated the ability of palmitoleic acid to sensitize the VRSA isolate (Fig. 2G and H). VRE strains have the same D-ala-D-ala precursor that is converted to D-ala-D-lac in the presence of vancomycin (1). Together, these data suggest that palmitoleic acid increases the rate of vancomycin killing and sensitizes vancomycin-resistant strains likely by outpacing the expression of vancomycin resistance genes.

Palmitoleic acid is a naturally occurring monounsaturated fatty acid found in macadamia nuts, sardines, and buckwheat oil (14), as well as in human serum and adipose tissue (15). Our group has shown that palmitoleic acid potentiates vancomycin by causing regions of increased fluidity (RIFs) in the membrane (7). We also showed that palmitoleic acid potentiated gentamicin efficacy against MRSA biofilms *in vitro* and in a diabetic wound infection model (9). The therapeutic potential of palmitoleic acid is also being examined for other indications (16, 17). Preclinical studies showed that oral delivery of palmitoleic acid can reduce inflammation by lowering the levels of pro-inflammatory cytokines and other inflammatory markers (16, 17). Additionally, oral delivery of palmitoleic acid has been shown to improve insulin sensitivity and glucose metabolism in mice with type 2 diabetes (18). Topical application of palmitoleic acid accelerated wound healing in an uninfected rat wound model (19). Some clinical trials are underway to explore the efficacy of palmitoleic acid in various therapeutic areas, including its ability to reduce inflammatory biomarkers and ameliorate insulin resistance in overweight or obese individuals (20). Despite the promise of these pre-clinical studies, an *in vitro* study suggested palmitoleic acid exerted toxicity to leukocytes at concentrations as low as 50 µM (21). However, the biological relevance of this finding is unclear given that the average serum concentration of palmitoleic acid in healthy adults far exceeds this at 133 µM (22). One possible explanation for this discrepancy is that unesterified fatty acids are highly (~99%) protein bound under physiological conditions, with a very small proportion of free fatty acids available to exert cytotoxic activity (23).

Although VRSA isolates are rare, they have been reported in diabetic patients with chronic infections (5), indicating a possible competitive advantage for VRSA in a diabetic environment. With the number of people with diabetes rising globally, the prevalence of VRSA strains may also increase. In this study, we have shown that palmitoleic acid can reverse vancomycin resistance in VRSA and VRE *in vitro*. Subsequent studies are required to examine the ability of topical palmitoleic acid to improve vancomycin therapy against VRSA and VRE wound infections. Additionally, oral delivery of this drug combination could potentially be used to target gut reservoirs of VRE.

### Bacterial strains and growth condition

VRSA strains (VRS1, VRS2, VRS4, VRS10, VRS11b, VRS13, BR-VRSA) and VRE strains (V587 and TX104) were obtained from BEI (10). CA-MRSA strain JE2 (24) and VRSA strains were grown in tryptic soy broth (TSB) at 37°C with continuous shaking at 225 rpm. VRE strains V587 and TX104 were grown in TSB at 37°C without shaking.

### *In vitro* killing assay

*In vitro* killing assays were carried out following the protocols previously described (7). Briefly, overnight cultures were diluted to 1:1,000 in fresh media, grown to ~1 × 10^8^ CFU/mL, and treated with palmitoleic acid (30 µg/mL) and/or vancomycin (50 µg/mL). After 6 h, bacterial cell aliquots were removed, pelleted, washed with PBS, serially diluted, and plated on tryptic soy agar for CFU enumeration. Statistical significance was determined as described in the figure legends. For induction of the *van* resistance genes, overnight cultures of JE2 or VRS1 were diluted to 1:1,000 in fresh TSB containing sub-MIC of vancomycin (0.25 µg/mL for JE2 or 16 µg/mL for VRS1) and grown to ~1 × 10^8^ CFU/mL of cells prior to challenge with palmitoleic acid (30 µg/mL) and/or vancomycin (50 µg/mL). Enumerating survivors was performed as described above. Data represent the mean value of *n =* 6 biological replicates ± standard deviation, and statistical significance was determined by unpaired student *t*-test.

### Minimum inhibitory concentration assay

MIC of vancomycin was determined by using the broth microdilution method. Briefly, bacterial strains were grown overnight in TSB and back diluted in fresh media. Approximately 5 × 10^5^ CFU were incubated various concentrations of vancomycin or palmitoleic acid in a 96-well plate (in biological triplicate). Plates were covered with Breathe-Easier sealing strips (Sigma) and incubated at 37°C without shaking for 24 h. The MIC was determined by the lowest concentration of the drug that prevented visible bacterial growth.

### Construction of transcriptional reporter of the vancomycin resistance genes

For the construction of a transcriptional reporter P*_vanH_-gfp* of the vancomycin resistance genes, the 244 bp sequence containing the *vanH* promoter (P*_vanH_*) was PCR amplified from genomic DNA of VRS2 and EcoRI/Xbal cloning sites were added using primers 5′-AGCTGAATTCGATGCTGGTGTCATCCA-3′ and 5′-GGCCTCTAGAATTAAGACCAACCCTTT-3′. The P*_vanH_* was inserted into EcoRI/Xbal site of pALC1434 plasmid upstream of *gfp* (25) to yield P*_vanH_-gfp*. Plasmid *P_pflB_-gfp* (26) was used used as a promoter-*gfp* control. Plasmids were first transformed into the restriction site-deficient *S. aureus* strain RN4220 (27) and ultimately into VRS1 and JE2. Transformed bacterial colonies were picked from TSA plates containing 10 µg/mL chloramphenicol and were confirmed by PCR.

### Quantifying induction of vancomycin resistance genes

JE2 and VRS1 containing the P*_vanH_-gfp* (vancomycin-resistant genes reporter) or P*_pflB_-gfp* (background control) plasmids were grown in the presence of 10 µg/mL chloramphenicol and with or without sub-MIC of (0.25 µg/mL for JE2 or 1 µg/mL for VRS1) vancomycin in a black clear flat bottom 96 well plate with lid at 37°C with continuous shaking inside a plate reader (SYNERGY H1, Biotek). At 30 min intervals, GFP fluorescence (excitation at 480 nm, emission at 510 nm) and optical density (wavelength 600 nm) were measured. To omit background fluorescence, GFP was expressed as (GFP-P*_vanH-gfp_* – GFP-P*_pflB-gfp_*)/OD- P*_vanH-gfp_*. Data represent the mean value of *n =* 6 biological replicates ± standard deviation, and statistical significance was determined by unpaired student *t*-test.

### Rate of killing assay

Overnight cultures of JE2 were diluted 1:1,000 in fresh media and grown to ~1 × 10^8^ CFU/mL. Cultures were treated with 30 µg/mL palmitoleic acid and/or 50 µg/mL vancomycin. At indicated times (10–120 min), aliquots were removed, washed with PBS, serially diluted, and plated on tryptic soy agar for CFU enumeration. The rate of killing (log_10_ CFU_tN_ − log_10_ CFU_t0_)/Time was determined using the linear range of the killing assay (indicated by the *R*_2_ value and vertical line in Fig. 1A through C). The mean of *n =* 6 biological replicates was shown, and statistical significance was determined by unpaired student *t*-test.

## Data Availability

Bacterial strains created in this study and raw data used to generate figures available upon request to the corresponding author.
